# A Study on Explosion Separation Technology of Carbon Fiber Reinforced Epoxy Resin-Based Composite Laminate

**DOI:** 10.3390/ma13163598

**Published:** 2020-08-14

**Authors:** Zhifu Wang, Meng Wang, Kunlun Hu, Zhiyuan Xia, Liubo Ma

**Affiliations:** School of Chemical Engineering, Anhui University of Science and Technology, Huainan 232001, China; Wzf15055411211@163.com (Z.W.); klhu@aust.edu.cn (K.H.); X1728435807@163.com (Z.X.); 18255466940@163.com (L.M.)

**Keywords:** aeroengine casing containment experiment, carbon fiber reinforced epoxy resin-based laminate, explosion separation technology

## Abstract

In an aeroengine casing containment experiment, in order to explode and separate unidirectional carbon fiber reinforced epoxy resin-based laminate, with uneven thickness, without excessive residual speed and fragment spattering, blades were subjected to three types of blasting and cutting pretests, including normal and lateral opening charge explosive tests on the laminate and linear charge-shaped jet cutting. The linear charge-shaped method was found to be the most suitable method for separating the laminate. The finite-element analysis program AUTODYN was used to simulate and optimize the effect of shaped jet cutting. When the explosive height of the shaped jet cutter was set to 90 mm, the laminate broke with the least number of fragments and the residual velocity of the plate was the smallest. At this time, we obtained the relationship between the total amount of explosive and the thickness of the composite plate when the composite plates of different thicknesses were just broken, and the rationality of the relationship was verified by experiments. The research method, in this paper, provides a reference scheme to design explosive separation composite materials in complex engineering environments.

## 1. Introduction

Compared with commonly used materials, such as metals and alloys, fiber composite materials have excellent properties, such as a light weight, high specific strength, high modulus, and high rigidity. Therefore, they have been used extensively in many fields, especially in aerospace [1,2,3]. The use of composite material has improved aircraft aeroelasticity and structural weight has been reduced, which has promoted aircraft structure modularization and lightweightedness. Therefore, the application of composite materials in military and civilian aircraft is expanding continuously. In addition to the use of composite materials in aircraft load-bearing structures, such materials have also entered the field of power-propulsion systems. The choice of material for helicopter propeller blades has changed from metal alloys to resin-based fiber composite materials. As a result, aeroengines have also entered the era of composite material.

Non-containment aeroengine accidents occur during operation when a rotor blade breaks suddenly during rotation and dangerous high-speed and high-energy debris flies through the casing. This debris can damage the aircraft cabin, fuel tank, hydraulic lines, and electrical control lines, which endangers flight safety and leads to serious air crashes. Therefore, it is of great significance to conduct aeroengine casing containment research [4]. Casing containment tests are used to verify the casing containment ability of rotor blades that fly out after breaking. In casing containment tests, blades must break at a specific position and speed without excessive residual speed and fragment splash. The explosion method is used to achieve blade separation and to limit excess power to the blade. Troshehenko [5] studied the fatigue crack growth of titanium alloy compressor rotor blades, determined the stress intensity factor of the test piece, and studied the blade and blade material fatigue crack growth. Warren [6] studied the stress state and fracture process of isotropic linear metal blocks after being subjected to quasi-static tensile stress. Xuan Haijun et al. [7] measured the rotor shaft trajectory and the load limit bearing on the ZUST6D high-speed rotating test bed at Zhejiang University. Andrew [8] used a gas cannon to perform ballistic impact tests on different composite plates and fan casings and compared the impact resistance properties of different materials. He also used three different structures of blades for impact tests. He found that when a pre-bent blade was used for the shooting test, a damage pattern was obtained that was similar to that of a rotating blade hitting a flat plate after flying off. Hu et al. [9] drilled holes and filled explosives in the center of the side of a titanium alloy composite plate and used experimental and numerical simulation methods to obtain the optimal ratio between the minimum wall thickness of the explosive separation member and the charge diameter. After that, Hu et al. [10] studied the explosive separation of plates with smaller thickness by prefabricating guide holes and guide grooves on the plate surface. Shihui Xiong [11] used AUTODYN finite element simulation software and proposed a three-dimensional (3D) finite element model of a pyrotechnic cutter to determine the influence of the explosive dynamic fracture process and the cutter blade acceleration distance on the cutting effect when the cutter was used to cut the wrapping-band connecting structures of carrier rockets.

At present, research on explosion separation has focused mainly on metal components, but as the aeroengine blade material change from metal alloys to carbon fiber reinforced epoxy resin-based composite materials, the blades have entered the era of composite materials. It is necessary to study the explosive cut-off of composite materials to meet the needs of aeroengine casing containment research in the era of composite materials. Composite materials are anisotropic materials, and their fracture mechanism differs from isotropic materials. The explosive cutting method that is applied to isotropic materials may not be applicable to composite materials. This work focused on unidirectional carbon fiber reinforced epoxy resin-based laminates with experiments and numerical simulation to study the explosive separation of laminates of different thicknesses. Blades of uneven thickness, such as those used in aeroengines, were exploded and separated in casing containment experiments to provide a reference solution to characterize explosive separation composite materials in complex engineering environments.

## 2. Determination of Test Method for Explosive Separation

### 2.1. Experimental Design

Unidirectional carbon fiber reinforced epoxy resin-based composite laminate was used. It was provided by Zhejiang University. This material uses carbon fiber as the reinforcing phase and epoxy resin as the matrix phase. The carbon fiber bundle is embedded in the resin in the same direction through autoclave or vacuum diversion. The unidirectional composite material forms after curing. The bundles are stacked at a staggered angle, and then cemented and solidified by resin to form a carbon fiber composite laminate.

To explode the unidirectional carbon fiber reinforced epoxy resin-based composite laminate and to meet the requirements of the containment experiment, three blasting and cutting pretests were carried out on the laminate. The tests included normal and lateral opening charge explosive tests on the composite plate and linear charge-shaped jet cutting, the blasting separation method that was most suitable was determined by comparing the separation effects of the three blasting cuts in the pretests. The experimental objects were 120 mm × 120 mm × 15 mm and 120 mm × 120 mm × 25 mm laminate. 

**Figure 1:**
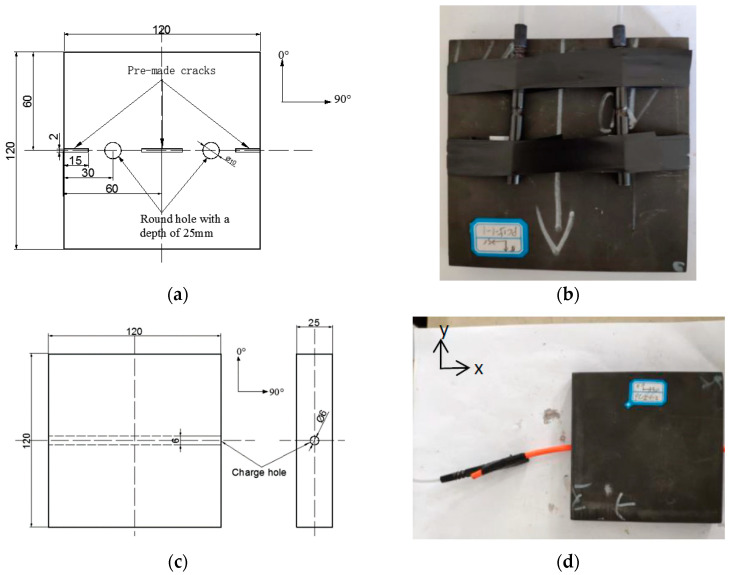
Schematic diagram and charge structure of the normal opening and lateral opening of the composite plate. (**a**) Schematic diagram of the normal opening of the composite plate; (**b**) Normal opening charge structure; (**c**) Schematic diagram of the lateral opening of the composite board; (**d**) Lateral opening charge structure.

The normal opening charge is to open two through 10 mm diameter holes in the middle of the laminate for charging and to make guide grooves along the 90° direction on both sides of the charging hole, as shown in Figure 1a. During the test, the end of the charging hole was closed with thin copper and desensitized RDX detonating cord was inserted into the charging hole. After charging was completed, the charging hole was sealed with paper, and the flanks of two nonel detonators of the same model were fixed with tape to the center of the two holes, as shown in Figure 1b.

The lateral opening charge was drilled in the thickness direction of the laminate. A 6 mm through-hole was drilled in the 90° direction of the flat plate for charging, as shown in Figure 1c. For charging and detonation convenience, a detonating cord that was 8 cm longer than the composite board was inserted into the lateral through-hole. The flanks of the detonator were fixed, and the detonating cord was extended with tape, as shown in Figure 1d.

**Figure 2:**
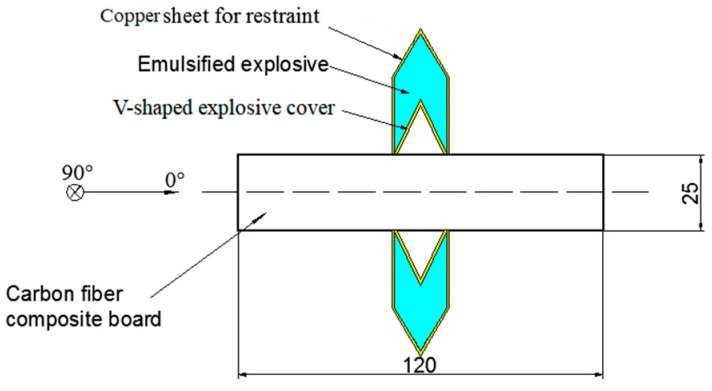
Schematic diagram of shaped energy jet cutting.

**Figure 3:**
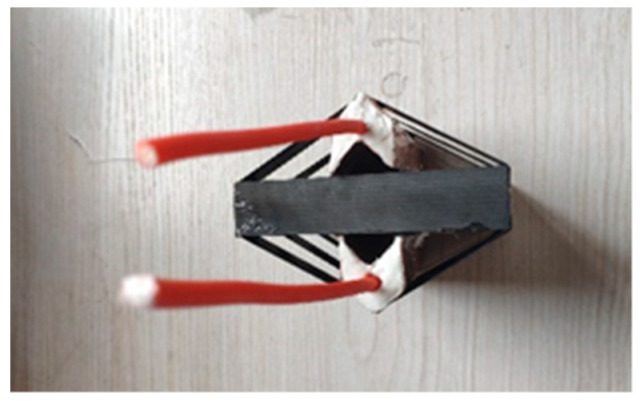
Shaped energy jet cutting charge structure.

The principle of linear shaped jet cutting is that after the charge desensitized RDX in the cutter was detonated, the product of explosion and shock wave squeeze the V-shaped explosive cover and push it to form a jet that rushes to the laminate and cuts it off. The schematic is shown in Figure 2. The shell of the cutter and the V-shaped explosive cover were made from 1 mm thick copper. The shell and explosive cover were attached with glue. The internal charge was desensitized RDX, and the port was closed with plasticine. The two cutters were fixed symmetrically on the front and back of the composite board and exploded by detonating cord, as shown in Figure 3.

### 2.2. Test Results and Discussion

**Figure 4:**
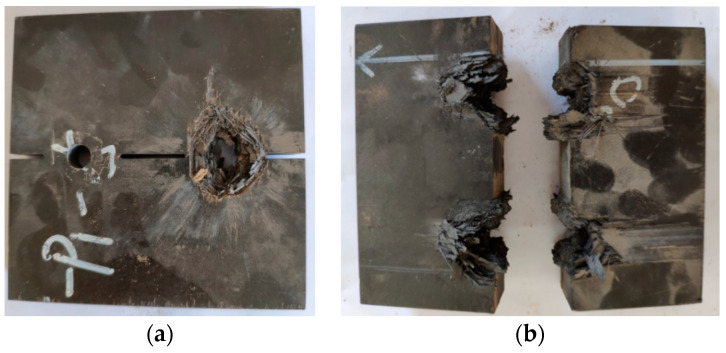
Normal opening charge blasting test results. (**a**) 15 mm; (**b**) 25 mm.

The explosion cut result of the charge in the normal opening is shown in Figure 4. One of the charge holes on the 15 mm thick laminate was not detonated because it was not attached tightly to the main detonation zone of the detonator, and therefore the composite board did not break. Two charge holes on the 25 mm thick laminate were detonated. Fibers around the charge holes were damaged, which damaged the fibers and matrix, and expanded the through-hole to the groove. The laminate broke, and the two broken parts were ~1.3 m apart. The laminate broke because, under this charging structure, the explosion generated a cylindrical shock wave, which propagated from the charging hole to the surroundings, and combined with explosion gas to expand the opening. The prefabricated guide groove expanded and the laminate broke. In [10], the thickness of the composite board was 8.5 mm, and the three normal opening methods did not successfully separate the composite board, because the thickness of the composite plate was too small, and a large amount of the gas generated by explosions escaped in the thickness direction and could not effectively act on the laminated plate. As the shock wave opening effect became smaller, it was difficult to expand the opening to the prefabricated guide groove, and the laminate would not broke. If the laminate is to be separated successfully, the prefabricated guide groove must be expanded to the edge of the charge hole, and at the same time, the plate cannot be broken in advance, which is too difficult to operate, and therefore, for small thickness laminates, this method is difficult to implement. The two fractured plates were 1.3 m apart in this pretest because the explosion shock wave provided a large additional lateral force to the fractured two plates in the fracture direction during the expansion and propagation, which resulted in a long distance after the composite plate was separated. It is difficult to meet the requirement that the laminate breaks only in the containment test without excessive residual speed and fragment splash.

**Figure 5:**
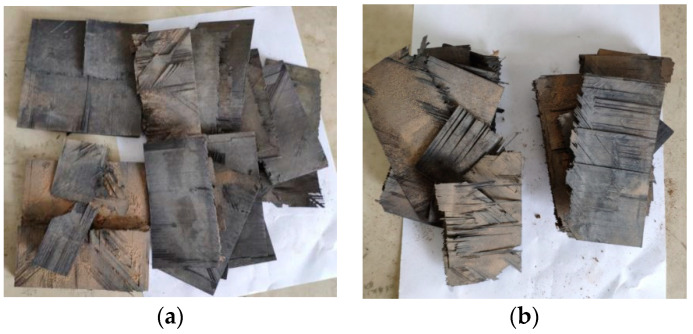
Results of lateral opening charge blasting test. (**a**) 15 mm; (**b**) 25 mm.

The results of the explosive cutting of the lateral opening charge are shown in Figure 5. Two laminates of different thicknesses were cut, but along the thickness direction, the laminate disintegrated because the huge explosive energy blew it into many pieces. The shock wave pressure that was generated by the explosion was greater than the Y-direction tensile strength of the laminate, which made the laminate fracture. However, this laminate was composed of many single-layer carbon fiber boards that had been superimposed by a specific method, and the tensile strength in the thickness direction was low. When the shock wave was transmitted from the inner hole of the plate to the interface between the plate and the air in the thickness direction, since the acoustic impedance of air was much smaller than that of laminates, most of the stress wave was reflected back to form tensile stress wave that was opposite to the propagation direction. The peak pressure of the tensile stress wave was greater than the tensile strength of the laminate in the thickness direction, therefore, the laminate disintegrated in the thickness direction. The experimental results did not meet the requirements of the containment test.

**Figure 6:**
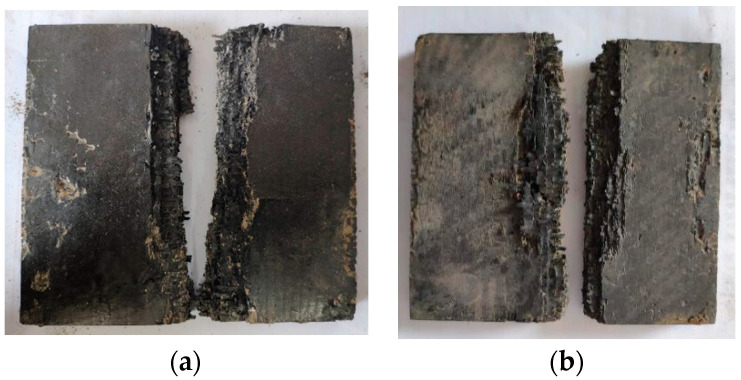
Results of shaped cutting test. (**a**) 15 mm; (**b**) 25 mm.

**Figure 7:**
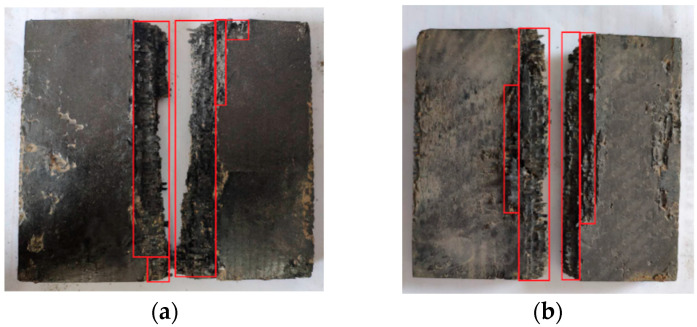
Schematic diagram of surface damage. (**a**) 15 mm; (**b**) 25 mm.

The results of the linear charge-shaped jet cutting are shown in Figure 6. Two laminates of different thicknesses were broken. The two parts of the 15 mm thick laminate are ~0.7 m apart, and the two parts of the 25 mm thick laminate are ~0.4 m apart. After the explosion, a certain degree of damage resulted on the surface of the incisions of the two laminates with different thicknesses. In Figure 7, the area enclosed by the red frame is the damage area of the plate after the laminate was cut. The damage area of the 15 mm thick plate was about 27.9% of the entire sample, and the damage area of the 25 mm thick plate was about 28.36% of the entire sample. This damage meant that a considerable number of additional fragments were produced. Fragments result because the explosion height is not set between the cutters and the laminate, and the jet reaches the laminate before it is formed sufficiently. Large lateral forces result during cutting, which result in a long distance between the two parts after the laminate is separated, causing a large degree of damage to the laminate surface.

The pretest results show that normal opening charge blasting cutting is not conducive to cutting thin laminate, whereas the broken board retains a higher speed, explosive cutting of the lateral opening charge, and the laminate is easy to delaminate. Therefore, the above two experimental methods do not meet the requirements of the containment experiment. After linear charge-shaped jet cutting, although damage occurs near the cut surface of the composite plate, and the fractured composite plate has a higher lateral velocity, the damage can be reduced by adjusting the explosion height and amount of explosive. Therefore, among the three cutting methods, linear charge-shaped jet cutting is the best way to separate the composite boards.

After the composite plate has been separated, additional fragments splash the least when the surface damage is a minimum, and the minimum lateral velocity of the broken plate occurs when the plate breaks under the minimum explosive amount. In Section 3, a numerical simulation method is described that is used to obtain the explosion height that can minimize the degree of surface damage to the composite plate and maximize the penetration depth of the jet (the minimum amount of explosive when cutting the composite plate of the same thickness) to optimize the linear charge-shaped jet cutting and to meet the requirement of the containment experiment that composite board is separated under the premise of an uneven blade thickness.

## 3. Numerical Simulation

### 3.1. Establishment of Model to Explore the Best Explosion Height

When linear charge-shaped jet cutting is performed, the materials that are used in the V-shaped explosive cover, the cone angle and the cutter height affect the cutting. Copper is the material of choice for V-shaped explosive cover, because copper has excellent comprehensive properties which include good plasticity, high density, sound velocity, and the ability to yield an extensible jet, as well as being inexpensive, and easy to manufacture. Among the many copper group materials, red copper produces the best shaped jet effect. Wang [12] confirmed through numerical simulation and experiment that a V-shaped explosive cover cone angle of 80° yielded a better cutting performance than cutters with other cone angles. Different optimal explosion heights occur for target plates of different materials and different types of explosives used by the cutter [13,14,15,16,17].

**Figure 8:**
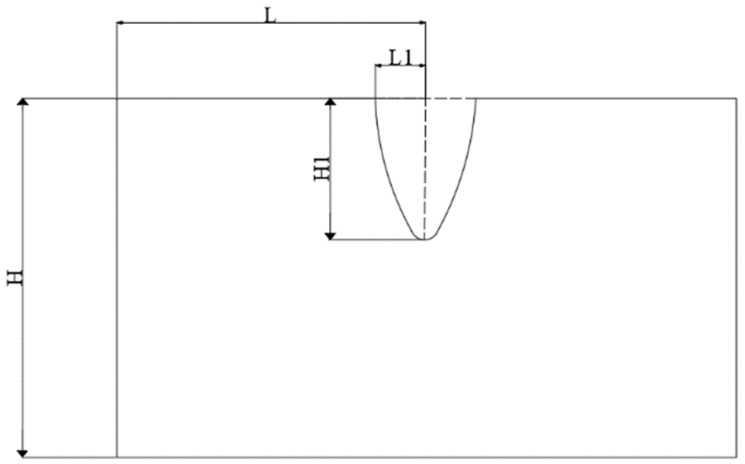
Schematic diagram of surface damage and penetration depth.

In summary, in this simulation, the shell and explosive cover were made from 1 mm thick red copper, the cone angle of the explosive cover was 80°, and desensitized RDX was used to make the linear charge-shaped jet cutter with unidirectional carbon fiber reinforced epoxy resin-based laminate as the target board. The aim was to find an explosion height which could get the largest jet penetration depth and smallest surface damage under the same explosive amount. Figure 8 shows that L1 is the length of the surface damage after the composite plate breaks, α = L1/L represents the degree of laminate surface damage, and H1 represents the depth of jet penetration.

During linear charge-shaped cutting, the process of crushing the drug cover, forming and stretching the jet, and penetrating the composite board is a large deformation movement with multimaterial interaction. The dynamic response of a material when subjected to an external load is characterized mainly by deformation, flow, and fracture. The description of its dynamic response is a complicated process, which is generally described by equations of state and constitutive equations. The equation of state of a material is a relational expression that is related to pressure, density, and some thermodynamic parameters (internal energy and temperature), which reflect the volumetric characteristics of the material. The constitutive equation reflects the bias strain of the material, which corresponds to the material strain, strain rate, internal energy, and grain size. The simulation used the AUTODYN module in ANSYS 17.0. The composite material used KFRP material in the program AUTODYN, and the calculation used the orthotropic constitutive model (ortho constitutive model), orthotropic yield strength model, and orthotropic softening failure model with the parameters shown in Table 1. The constitutive red copper model used the Johnson-Cook model, the equation of state used the Gruneisen Equation, and the parameters shown in Table 2. The constitutive model of explosives used the high explosive burn model, the equation of state used the JWL equation [18], and the parameters shown in Table 3.

**Figure 9:**
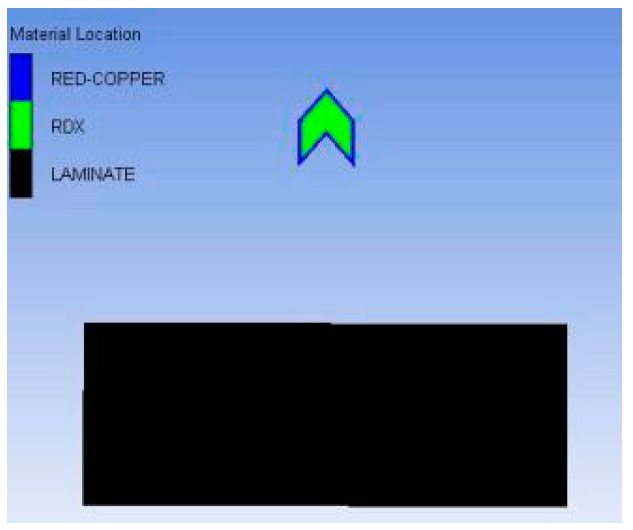
Numerical model diagram.

Most numerical simulation research on linear charge-shaped jet cutting uses the Langrage algorithm. Because air is considered in the Langrage algorithm, the contact surface between air and the jet needs to be defined. During the jet forming process, a negative volume appears because of the large deformation of the air grid, which causes the calculation to stop halfway. To ensure continuity and accuracy of the calculation, the sph algorithm was used for calculation. This simulation was used to explore the best explosion height, therefore, the cutting device was arranged only on one side of the target plate with 35 g explosive; a 45 mm thick target plate; an explosion height of 30, 40, 50, 60, 70, 80, 90, 100, and 110 mm; and eight sets of models. Figure 9 shows a numerical modeling diagram, where the blue part is red copper, the green part is RDX (explosive), and the black part is unidirectional carbon fiber reinforced epoxy resin-based composite laminate. We imposed a fixed boundary on the bottom of the laminate in the direction perpendicular to the jet, with a velocity of zero.

### 3.2. Calculation Results and Analysis

**Figure 10:**
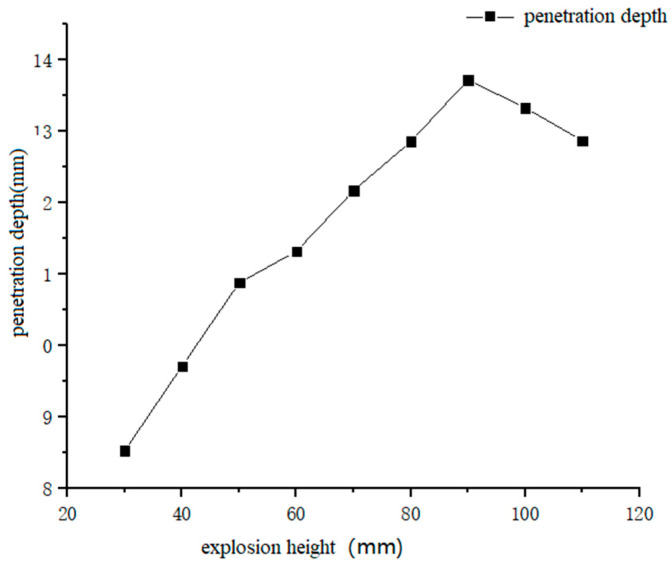
Curves of penetration depth that correspond to different explosion heights.

**Figure 11:**
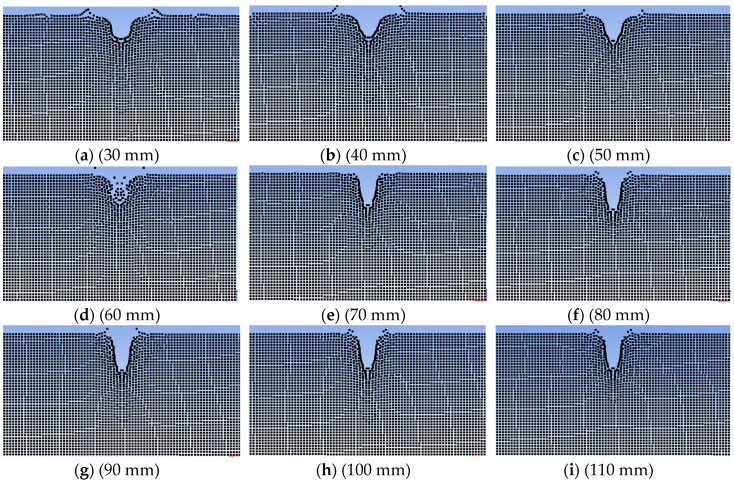
(**a**–**i**) Simulation calculation results for nine high explosion conditions.

**Figure 12:**
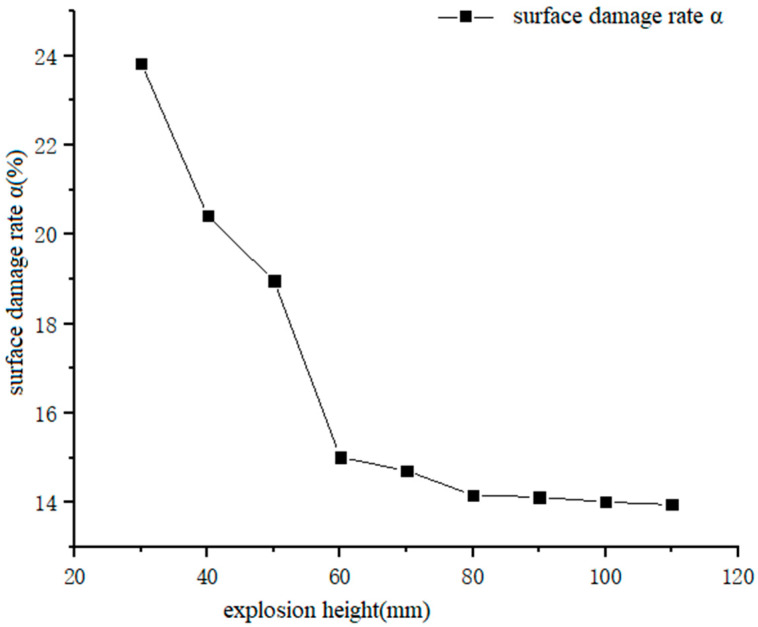
Curves of α data that correspond to different explosion heights.

Figure 10 shows the data curve of penetration depth as a function of explosion height. Combined with Figure 11, it can be seen that with an increase in explosion height, the penetration depth increased from 8.53 mm at an explosion height of 30 mm, to 13.72 mm at an explosion height of 90 mm, and then decreased to 12.87 mm at an explosion height of 110 mm. Figure 12 shows the data curve for the degree of surface damage α with explosion height. Combined with Figure 11, it can be seen that with an increase in explosion height, the degree of surface damage α decreased. When the explosion height increased to 80 mm, the decrease rate of α tended to be gentle, and was maintained at ~13.9%. When the explosion height was 90 mm, the jet was fully formed, the velocity of the jet head reached a maximum, and the body of the jet pestle was basically unchanged. The penetration depth of the jet reached a maximum at this point for the same amount of explosive, and damage of the pestle body to the cut surface of the laminate was minimized.

For a shell and an explosive cover from 1 mm thick red copper, an explosive cover cone angle of 80°, and a shaped jet cutter from desensitized RDX for explosive filling, for cutting of unidirectional carbon fiber reinforced epoxy resin-based laminate, the best explosion height was 90 mm. Under this working condition, laminates of different thicknesses were cut by adjusting the amounts of explosives, so that the laminates of different thicknesses could just break. When the laminate just broke, the relationship between the laminate thickness and the amount of charge was obtained. Therefore, the carbon fiber epoxy resin laminate with uneven thickness used in the aeroengine casing containment experiment could meet the requirements of the containment experiment when it was exploded and separated.

### 3.3. Establishment of a Numerical Model to Explore the Relationship between Laminated Plate Thickness and Amount of Explosive When the Laminated Plate at the Optimal Explosion Height Just Broke

**Figure 13:**
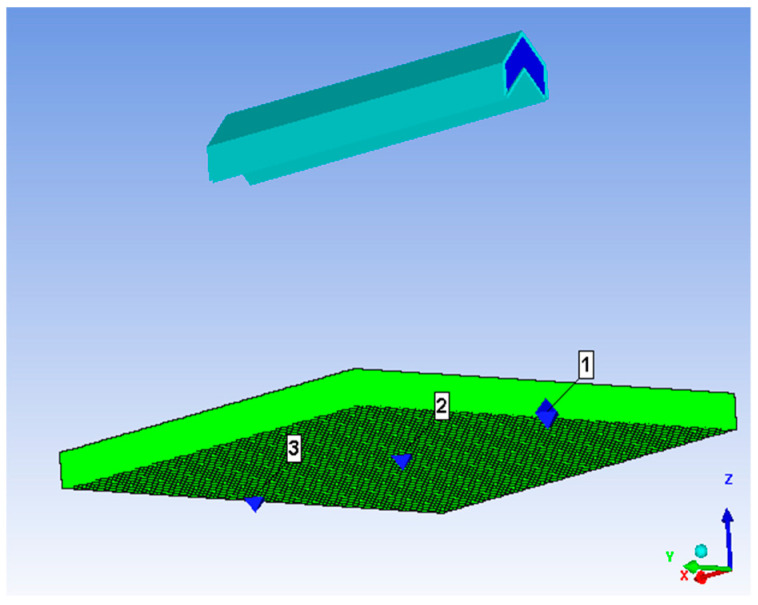
Numerical simulation model of linear charge-shaped jet cutting.

The laminate model length was set to 120 mm, the width was set to 120 mm, and the thickness was increased from 15 mm by 2 mm to 25 mm, with six model sets. The cutter length was set to 120 mm and the width was set to 15 mm. The amount of explosive was adjusted by adjusting the shell height. The cutter was placed symmetrically on the front and back of the laminate. Because the model was symmetrical along the thickness direction, to save calculation time, the half-plane symmetric model was used for modeling [19,20], and the default boundary condition of the software was the symmetric boundary condition. The simulation model is shown in Figure 13.

Carbon fiber reinforced epoxy resin-based laminates are anisotropic materials. The load prediction is much more difficult for composite structures than for metallic shells, beams, and plates [21], and their damage must be considered for the material tensile and compressive strength. Therefore, the Tsai-Wu tensor strength theory [22] was used to judge whether the single-layer material was damaged. One to three monitoring points were placed in the middle of the laminate thickness, and laminate fracture was judged by judging the destruction of each monitoring point when the jet reached the middle of the laminate. After the calculation was completed, the stress values at these points could be observed through the AUTODYN software and substituted into the Tsai-Wu tensor criterion to determine the failure state at these points, so that the fracture of the laminate when the jet reached the middle of the laminate could be judged.

### 3.4. Calculation Results and Analysis

First of all, the laminate thickness was set to 15 mm, and the amount of charge was adjusted by continuous adjustment of the shell height in the model to simulate the test. When the amount of explosive was too high, the height of the outer shell of the cutter was reduced. When the amount of explosive was insufficient, the height of the outer shell was increased, and the desensitized RDX was filled. The simulation was started first from the height of the larger outer shell, and then the range was reduced by the dichotomy method. When the height of the outer shell was 16.9 mm, according to the Tsai-Wu tensor criterion, the F-value of monitoring Points 1, 2, and 3 were 1.0076, 1.00485, and 1.00588, respectively. The laminate was broken, and the corresponding explosive consumption was 18.652 g per side. The data are shown in Table 4. Tsai-Wu tensor criterion polynomial:

(1) $$F=F_{1}\sigma_{1}+F_{2}\sigma_{2}+F_{11}\sigma_{1}{}^{2}+F_{22}\sigma_{2}{}^{2}+F_{66}\tau_{12}{}^{2}+2F_{12}\sigma_{1}\sigma_{2}$$

where $F_{1}=\frac{1}{X_{t}}-\frac{1}{X_{c}}={1.12873\times 10}^{-4}{\;\mathrm{MPa}}^{-1}$, $F_{2}=\frac{1}{Y_{t}}-\frac{1}{Y_{c}}=-{1.7459\times 10}^{-3}{\;\mathrm{MPa}}^{-1}$, $F_{11}=\frac{1}{X_{t}X_{c}}={2.1917\times 10}^{-6}{\;\mathrm{MPa}}^{-2}$,$F_{22}=\frac{1}{Y_{t}Y_{c}}={2.90978\times 10}^{-6}{\;\mathrm{MPa}}^{-2}$, $F_{66}=\frac{1}{s^{2}}={1.68663\times 10}^{-4}{\;\mathrm{MPa}}^{{}_{-2}}$, and $F_{12}=-\frac{1}{2}\sqrt{F_{11}F_{22}}=-{1.597\times 10}^{-6}{\;\mathrm{MPa}}^{-2}$.

**Figure 14:**
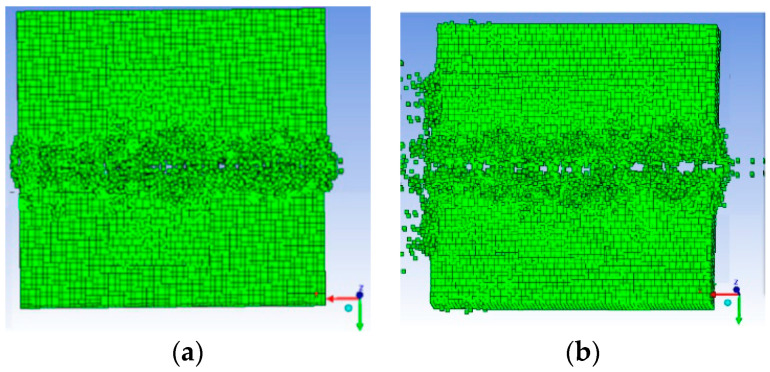
Simulation effect after explosion. (**a**) Laminates tend to separate exactly, (**b**) Laminate is broken.

Figure 14 is a simulation effect diagram after the explosion, in which (a) is the picture when the laminate just broke, (b) is the picture when the laminate was completely broken. Table 5 shows the amount of desensitized RDX in the cutter on each side of the laminate when the laminates of each thickness have just been cut. The data were fitted numerically to obtain the relationship between the laminate thickness and the amounts of explosives on each side of the cutter when the laminate just broke under the above working condition, as shown by Equation (2). Therefore, the relationship between the laminate thickness and the total amount of explosives is given by Equation (3):

(2) $$Y=-1.0466+1.33386x-0.00118x^{2}$$

(3) $$Y=-2.0932+2.66772x-0.00236x^{2}$$

where *y* is the total amount of passivated RDX and *x* is the thickness of the corresponding laminate. The suitability of Equation (3) was verified experimentally.

## 4. Experimental Verification

**Figure 15:**
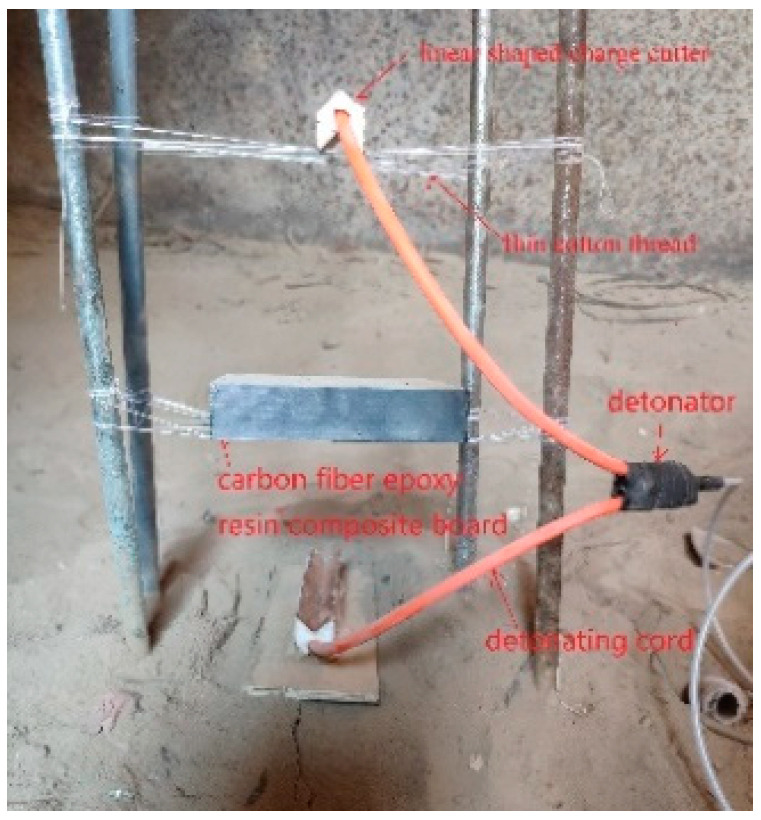
Experimental device.

To provide a factual basis for the numerical simulation results, when the explosion height was set to 90 mm, for laminate thicknesses of 15, 17, 19, 21, 23, and 25 mm, linear charge-shaped jet cutting was carried out by using the explosive amount in the simulation results. As shown in Figure 15, the cutter was made according to the simulated parameters, and the distance between the cutter and the laminate was set according to the best blast height obtained in Section 3.1. The experimental device was put into a large explosion-proof warehouse and detonated with a detonator.

**Figure 16:**
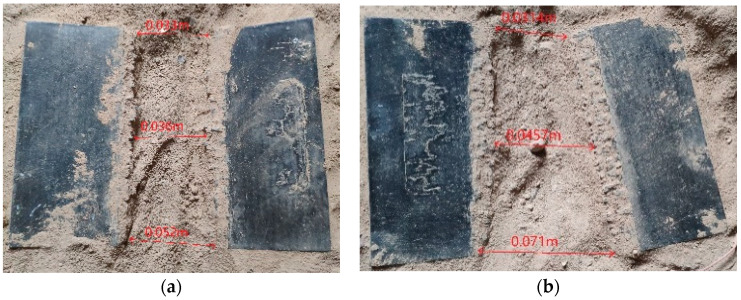
Schematic diagram of the distance between the two parts of the broken board. (**a**) 15 mm; (**b**) 25 mm.

Figure 16 shows a schematic diagram of the distance between the two parts of the broken board after the 15 and 25 mm thick laminates were cut. After the explosion, the cuts of the two broken plates were not aligned, so the distances between the upper, middle, and lower parts of the two broken plates were measured, and the average value was taken as the distance between the broken plates.

**Figure 17:**
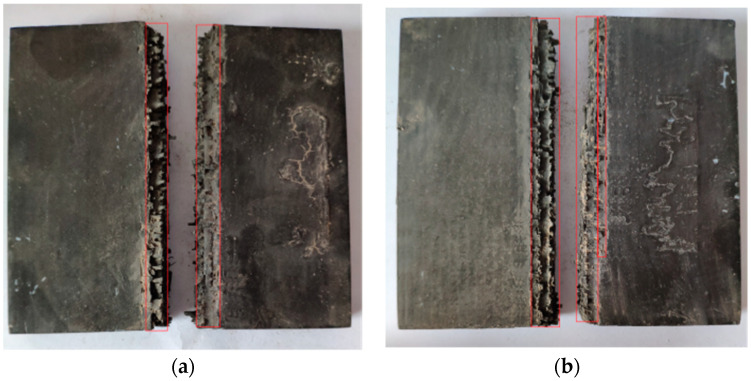
Schematic diagram of surface damage of laminated board. (**a**) 15 mm; (**b**) 25 mm.

According to Table 6, the distance between the broken plates after the 15 mm thick laminate broke was 0.04 m, and the distance between the broken plates after the 25 mm thick laminate broke was 0.049 m. When the laminate was broken, the explosion provided a small lateral velocity to the broken plate. The broken plate almost moved freely, and the plate was close to breakage. Figure 17 shows a schematic diagram of the surface damage after cutting 15 and 25 mm thick laminates. The red frame area was the damaged laminate area. Compared with the results of the energy-sharing cutting experiment in the pretest, the degree of surface damage on the broken board after laminate fracture was reduced significantly, which met the requirements of the containment experiment.

Table 7 compares the simulation calculation and verification experimental results. The simulation results represent when the laminated plate happened to break, but there was a falling process of the laminated plates that occurred in the experiment. Thus, there was a distance of several centimeters between the broken plates. The maximum and minimum relative errors between the numerical simulation of α and the test value were 9.02% and 1.98%, respectively, and both were within 10%. The numerical simulation results agreed well with the test conditions. Therefore, for unidirectional carbon fiber reinforced epoxy resin-based laminates of a certain thickness, the relationship between the laminate thickness and the total amount of explosives from the simulation results is reasonable, and it can be used in experiments of aeroengine casing containment.

## 5. Conclusions

In the aeroengine casing containment experiment, the goal was to make the unidirectional carbon fiber reinforced epoxy resin-based laminate, with uneven thickness, explode and separate without an excessive residual speed and fragment spattering. Experiments and numerical simulation were used to study the explosive separation of laminates with different thicknesses. The following conclusions were obtained:

(1) For unidirectional carbon fiber reinforced epoxy resin-based laminates, of the three explosive separation methods (normal opening charge, lateral opening charge, and linear charge-shaped jet cutting), linear charge-shaped jet cutting was the most suitable explosive separation method.
(2) For cutting of the unidirectional carbon fiber reinforced epoxy resin-based laminate, the best explosion height was 90 mm. Under this working condition, the relationship between the total amount of explosive and the laminate thickness was $Y=-2.0932+2.66772x-0.00236x^{2}$, no excessive residual velocity and fragment splash occurred after the laminate was broken. It met the requirements of the containment experiment.

## Figures and Tables

**Table 1:** Performance parameters of carbon fiber epoxy resin composites.

**Young Modulus (MPa)**			**Shear Modulus (MPa)**			**Poisson Ratio**		
*E*_1_	*E*_2_	*E*_3_	*G*_1_	*G*_2_	*G*_3_	*v*_1_	*v*_2_	*v*_3_
61.66	58.18	9.43	3.65	3.22	4.19	0.065	0.451	0.543
**Tensile Strength (MPa)**			**Compression Strength (MPa)**			**Shear Strength (MPa)**		
*X*_t_	*Y*_t_	*Z*_t_	*X*_c_	*Y*_c_	*Z*_c_	*S*_xy_	*S*_xz_	*S*_yz_
1894	617	92.4	2409	557	49.5	77	61.8	49.5

**Table 2:** Red copper calculation parameters.

Material	Density *ρ/*(g·cm^−3^)	Speed of Sound in Material (m·s^−^^1^)	Gruneisen Parameter S_1_	Gruneisen Parameter S_2_	Gruneisen Parameter S_3_	Gruneisen Parameter γ_0_
Red copper	8.96	4750	3.80	2.74	0.13	1.35

**Table 3:** Calculation parameters of desensitized RDX.

Density *ρ*/(g·cm^−3^*)*	Speed of Explosion *D/*(m·s^−1^)	CJ Pressure P_cj_/(GPa)	Initial Internal Energy *E*_0_/(GJ·m^−3^)	JWL Coefficient *A*/(GPa)	JWL Coefficient *B*/(GPa)	JWL Coefficient *R*_1_	JWL Coefficient *R*_2_	JWL Coefficient ω
1.786	8720	33.59	14.801	210	475.4	4.3	1.3	0.34

**Table 4:** Stress values and F values of monitoring points.

Stress	1^#^	2^#^	3^#^
*σ*_1_/MPa	−887.7	−638	−546.4
*σ*_2_/MPa	−472.1	−512	−482.0
*τ*_12_/MPa	94.4	82.52	79.1
F	1.0076	1.00485	1.00047

**Table 5:** Amount of charge per side when the same thickness plate just broke.

Amount of Charge per Side When the Same Thickness Plate Just Broke						
Laminate thickness/mm	15	17	19	21	23	25
Amount of charge per side/g	18.652	21.362	23.876	26.407	28.976	31.589

**Table 6:** Cut-off experimental data comparison.

Results of Shaped Cutting Experiment in Preliminary Experiments and Confirmatory Experiment	15 mm Thick Laminate		25 mm Thick Laminate	
	The Distance between the Broken Plates/m	Approximate Percentage of the Area of the Red Frame to the Surface Area of the Laminate/%	The Distance between the Broken Plates/m	Approximate Percentage of the Area of the Red Frame to the Surface Area of the Laminate/%
Results of shaped cutting experiment in preliminary experiments	0.7	27.9	0.4	28.36
Results of confirmatory experiment	0.04	13.8	0.049	15.35

**Table 7:** Comparison of simulation calculation and verification experimental results.

Laminate Thickness/mm	Simulation Calculation and Verification Experiment	15	17	19	21	23	25
The distance between the broken plates/m	simulation calculation	0.000	0.000	0.000	0.000	0.000	0.000
	verification experiment	0.040	0.041	0.035	0.039	0.053	0.049
α/%	simulation calculation	13.2	13.65	13.83	13.63	14.02	14.27
	verification experiment	13.8	13.92	14.25	14.86	14.66	15.38
	Difference/%	4.54	1.98	3.03	9.02	4.56	7.78
